# Persistence and innovation effects in genetic and environmental factors in negative emotionality during infancy: A twin study

**DOI:** 10.1371/journal.pone.0176601

**Published:** 2017-04-27

**Authors:** Lyndall Schumann, Michel Boivin, Stéphane Paquin, Eric Lacourse, Mara Brendgen, Frank Vitaro, Ginette Dionne, Richard E. Tremblay, Linda Booij

**Affiliations:** 1 Department of Psychology, Queen’s University, Kingston, Canada; 2 School of Psychology, University of Laval, Quebec, Canada; 3 Institute of Genetic, Neurobiological and Social Foundations of Child Development, Tomsk State University, Tomsk, Russian Federation; 4 Department of Sociology, University of Montreal, Montreal, Canada; 5 CHU Sainte-Justine Research Centre, Montreal, Canada; 6 Department of Psychology, UQAM, Montreal, Canada; 7 Department of Psycho-education, University of Montreal, Montreal, Canada; 8 Department of Pediatrics and Psychology, University of Montreal, Montreal, Canada; 9 School of Public Health, Physiotherapy and Population Science, University College Dublin, Dublin, Ireland; 10 Department of Psychology, Concordia University, Montreal, Canada; Vanderbilt University, UNITED STATES

## Abstract

**Background:**

Difficult temperament in infancy is a risk factor for forms of later internalizing and externalizing psychopathology, including depression and anxiety. A better understanding of the roots of difficult temperament requires assessment of its early development with a genetically informative design. The goal of this study was to estimate genetic and environmental contributions to individual differences in infant negative emotionality, their persistence over time and their influences on stability between 5 and 18 months of age.

**Method:**

Participants were 244 monozygotic and 394 dizygotic twin pairs (49.7% male) recruited from birth. Mothers rated their twins for negative emotionality at 5 and 18 months. Longitudinal analysis of stability and innovation between the two time points was performed in Mplus.

**Results:**

There were substantial and similar heritability (approximately 31%) and shared environmental (57.3%) contributions to negative emotionality at both 5 and 18 months. The trait’s interindividual stability across time was both genetically- and environmentally- mediated. Evidence of innovative effects (i.e., variance at 18 months independent from variance at 5 months) indicated that negative emotionality is developmentally dynamic and affected by persistent and new genetic and environmental factors at 18 months.

**Conclusions:**

In the first two years of life, ongoing genetic and environmental influences support temperamental negative emotionality but new genetic and environmental factors also indicate dynamic change of those factors across time. A better understanding of the source and timing of factors on temperament in early development, and role of sex, could improve efforts to prevent related psychopathology.

## Introduction

Temperament refers to the individual differences in behavioural style and emotional functioning that appear in early infancy. Investigation of the biological basis of temperament with twins has provided evidence supporting the moderate heritability of individual differences in temperament [1–4]. Heritability refers to the proportion of variation in a trait that is attributed to genetic variation among individuals. Twin studies provide a framework from which to estimate heritable contributions by comparing observed phenotypic variability between pairs of monozygotic (MZ) twins, who share 100% of their genes, and dizygotic (DZ) twins, who on average share 50% of their genetic endowment. These studies also reveal that variability in temperament is also accounted for by shared (i.e., siblings sharing the same home) and non-shared (i.e., siblings interacting with different peers) environmental factors [2,4,5]. However, little is known about how key aspects of temperament, such as tendency toward negative emotionality, develop in the first two years of life, and whether the same or new genetic or environmental influences play a role in this development. A deeper understanding of the persistence and innovation of genetic and environmental sources of variance could help alleviate potential risks posed by difficult temperament in early life.

Longitudinal studies suggest that a difficult temperament may signal vulnerability for the development of anxious and depressive symptomatology in children [5–8]. Difficult temperament refers a constellation of behavioural tendencies, including unadaptability, lack of soothability, and negative emotionality. In the present paper, we focus on negative emotionality, defined as a disposition to experience negative emotional states. Expressed as an externalized response motivated by frustration and/or a lack of stimulation, and manifested in infants through fussiness, crying, and negative emotional behaviour, this dimension of difficult temperament has been documented as a risk factor for later psychopathology [9–11].

Negative emotionality is indeed one key temperamental characteristic common to both externalizing and internalizing disorders, most notably, depression and anxiety [7,11,12]. Furthermore, infant negative reactivity has been shown to predict [pre)frontal cortical morphology in adulthood [13]. Accordingly, negative emotionality may be a marker of a developmental pathway of psychopathology. Children’s heritable difficultness has also been shown to evoke a negative parenting response in mothers [14,15]. Thus, persistent high negative emotionality may interact with parenting and increase the risk of later psychopathology and adverse outcomes [16,17]. Despite its importance, the early development of negative emotionality, including its qualitative and quantitative sex differences [underlying contributions to variation), remains poorly documented.

The origins of child temperament have been the object of close attention in the 1980s and 1990s [4,18–21]. Genetic and environmental analyses of negative emotionality highlighted a substantial role of genetic factors [2,5,9,22–26]. Estimates of heritability (*h*^2^) typically ranged from 13% to 62%, with most of the estimates falling between 40% and 48% [2,3,24] Two cross-sectional studies investigated negative emotionality/affectivity in toddlers and pre-schoolers (55 monozygotic, or MZ pairs and 65 dizygotic, or DZ pairs aged 33–99 months; [2]), as well as in infants (121 MZ pairs and 181 DZ pairs aged 3–16 months old;[18]). A third cross-sectional study explored negative emotionality (used interchangeably with infant difficultness) in 865 infant twins (zygosity determined using probability estimation) ranging in age from 1 to 32 months born in Puerto Rico [5]. All three studies were based on parent reports and found substantial additive genetic and non-shared environmental, but no shared environmental contributions to negative emotionality. The proportion of variability in negative emotionality attributed to genetic effects was 42% in the toddler and preschool sample [2], 64% in infancy [27], and 75.2% overall from birth to 32 months [5]. Sex differences in the models were either not investigated [2] or not significant [5]. There was, however, a contrast effect contribution (B; possibly stemming from a bias from mother’s rating, leading to a greater difference between them) found in the 2005 study performed by Silberg and colleagues, thought to magnify the intra-pair differences. However, all three studies were cross-sectional in design and thus do not help us understand the stability and change in the genetic and environmental sources of these traits.

Furthermore, we know little about the change and persistence and innovation of negative emotionality’s heritability in infancy, a period during which many brain changes occur [28]. No studies known to the authors have tested an ACE model (the additive genetic, shared environment, and unique environment contributions) with sex differences and persistence and innovation of these contributions to negative emotionality, in populations under the age of three.

Operationalization of negative emotionality may also differ based on how it has been assessed. The method of assessment, by care-giver report or laboratory observation, can have an impact on the estimates of gene-environment contribution to temperament [29]. For example, parent reports tend to yield higher heritability estimates [29]. Parent reports of temperament are also more likely influenced by rater bias that tends to exaggerate the difference among co-twins [29,30]. However, other studies have found that parent report and observation are convergent [31], and have been found equally good at predicting related behaviour in different settings [32].

Only a few longitudinal twin studies have documented the genetic and environmental contributions to negative emotionality in toddlers [9,20,30]. Rhee et al. [9] assessed negative emotionality through behavioural observations in laboratory tasks at 14, 20, and 24 months (224 MZ pairs and 179 DZ pairs), and found heritability estimates of 62% at 14 months, 29% at 20 months and lower still at 24 months (13%). Conversely, the magnitude of shared environmental contribution increased from 5% to 51% over the same time period. No sex differences were found at any time point. Similarly, Saudino and Cherney [30] found that the heritability of parent-reported negative emotionality increased from 14 to 20 months (37% to 47%), and then decreased from 20 to 36 months (47% to 11%). The role of the non-shared environment followed the opposite pattern (initially decreasing over 14 to 20 months, then increasing from 20 to 36 months). However, neither study provided any information about the genetic and environmental contributions before 12 months of age or their persistence and/or change up to this point.

A few longitudinal twin studies have provided evidence that stability in certain traits and behavior patterns (activity, affect/extraversion, behavioural inhibition, disregard for rules, physical aggression), is substantially accounted for by genetic factors [29,30,33,34], while at the same time indicating that the genetic effects are developmentally dynamic, i.e. varying over time, with previously inactive genes coming online (genetic innovation; see [33, 34, 35]). Additionally, environment factors appeared to drive some of the change in temperament during this time [29,34,36]. It has been postulated that genetic factors may be largely responsible for continuity in temperament, whereas environmental factors are largely responsible for change in temperament [29]. Such findings have been documented (see [33 and 34]), but mainly during toddlerhood. However, genetic and environmental factors may operate or develop differently according to age- or sex-specific genetic innovations, occurring in early childhood [2,37]. So far, knowledge regarding continuity and change of genetic and environment contributions to negative emotionality’s in infancy is limited.

The goal of the present study was to use a longitudinal study of twins to estimate persistence and innovation in genetic and environmental contributions to negative emotionality between 5 and 18 months. We also explored sex differences. Sex differences in negative emotionality heritability have yielded mixed results, with some studies finding no significant sex differences [5,9]. Other studies have found that negative emotionality explains genetic influences on depression/conduct disorder comorbidity for boys more than girls (in children and adolescents; [23]), or significantly higher correlations between same sex, dizygotic (DZ) twins than opposite sex DZ twins [2]. The study aimed to investigate these in a large longitudinal cohort of twins (244 monozygotic, 200 same-sex dizygotic, and 194 opposite-sex dizygotic twin pairs) with measurements at 5 and 18 months.

## Method

### Participants

The participating twins were recruited at birth in the province of Quebec (Canada) between November 1995 and July 1998, as part of the Quebec Study of Newborn Twins, a longitudinal cohort study in newborn twins in Quebec [38]. The original study included 1324 children (662 twin pairs) at 5 months whose parents agreed to participate and for whom zygosity was determined. Participants were 662 boys and 662 girls. Zygosity was assessed using the Zygosity Questionnaire for Young Twins, and confirmed through DNA testing in 30% of the sample [15,39]. The sample was representative of Quebec in terms of family characteristics, with very similar parental education, yearly household income, age of parents at child’s birth, and marital status. Twin pairs were excluded if they did not have available data on negative emotionality for at least one twin at either 5 or 18 months. Demographic characteristics (see Table 1) for the sample used (N = 638 pairs) were comparable to the original sample (N = 662 pairs) in terms of infant’s sex, age of mother at birth, days premature, mother’s education level and family composition at both time points. The sample did differ for weight at birth and mother’s ethnicity.

**Table 1:** Descriptive statistics.

Demographic Characteristic	Sample used for analyses (N = 638 pairs)^1^	With missing on negative emotionality (N = 24 pairs)^2^		
	Mean or percentage (SD)	Mean or percentage (SD)	T or chi-square	Sig.
Girl* (%)	50.3	41.7	1.4	.24
Age of mother at twin pairs birth (years)	30.4 (4.8)	30.5 (5.1)	.05	.96
Days premature (days)	29.0 (17.6)	23.9 (22.7)	-.8	.41
Weight at birth* (kg)	2.44 (.56)	2.72 (.60)	2.8	.01
Mother has obtained secondary school diploma (%)	83.1	70.6	1.8	.18
Race/Ethnicity of mother				
White/Caucasian (%)	88.1	58.8	12.7	<.01
Family Status				
Families with less than 20 000$CAD/year (%)	17.9	35.3	3.4	.07
Twin pairs living with both biological parents at 5 months (%)	92.3	85.0	1.5	.23
Twin pairs living with both biological parents at 18 months (%)	90.0	85.0	.53	.47

Note: Means were compared with t-test and percentages with chi-squares.

*Values compared at the individual level, all other comparisons were at the family level.

^1^ Actual N varies from 493 to 638 pairs because of missing data on demographic characteristics.

^2^ Actual N varies from 8 to 24 pairs because of missing data on demographic characteristics.

### Ethical standards

All procedures contributing to this work complied with the ethical standards of the relevant national and institutional committees on human experimentation and with the Helsinki Declaration of 1975, as revised in 2008. Written informed consent was obtained from mothers. Mothers and researchers both signed the consent form. The appropriate Ethics board (Sainte-Justine Hospital) approved the studies/consent procedures.

### Measures

Children’s behaviour was assessed with the Bates Infant Characteristics Questionnaire [6] at 5 and 18 months during home visits. The mother of each twin pair filled out a 37-item checklist for one twin during the visit, and filled out another for the second child two weeks after.

#### Negative emotionality scale

The negative emotionality scale was composed of seven items from the Bates Infant Characteristics Questionnaire (item composition similar to the Fussy/difficult scale). The measure of negative emotionality has been used in the past to assess infant trait difficultness, anger, and fussiness [9,10]. The items measured how often the baby was fussy per day, amount of fuss/crying, intensity of protest, how easily the baby became upset, amount of attention the baby requires, how often the baby plays with self when alone, and overall degree of difficulty the baby presents for the parent on a seven-point scale. A score of 1 represented low negative emotionality and a score of 7 represented high negative emotionality. An average of these seven negative emotionality items was used in the analyses. Cronbach’s alpha for the sample included in the present analyses was .80 for the mother’s negative emotionality ratings at age 5 months and .77 for mother’s negative emotionality ratings at age 18 months. Phenotypic stability was computed to be *r* = .50 for boys and *r* = .51 for girls, indicating moderate stability in the trait from 5 to 18 months. Examination of the distribution demonstrated that the negative emotionality scale was normally distributed.

### Data analyses

The relative contributions of genetic and environmental factors to negative emotionality were determined using structural equation models based on the biometric model [40,41]. First, sex-limited cross-sectional models were fit at 5 months and 18 months where we explored both qualitative and quantitative sex differences. Second, a sex-limited multivariate correlational model [42,43] was used to estimate the additive genetic, shared environment and non-shared environment components of negative emotionality at 5 and 18 months. To interpret the results in terms of innovation of genetic and environmental influences, we transformed the correlation matrix as if we executed a Cholesky decomposition using classical algebra. The rationale for such an approach is further detailed in the description of our longitudinal model.

#### Univariate models at 5 and 18 months

Estimates of the additive genetic (A), dominant genetic (D), shared environment (C), and non-shared environment (E) components were based on comparison of the theoretical relationship between MZ or DZ twins reared together and the measured concordance between twins in a pair. The combined, girl, and boy MZ / DZ correlation matrices with means and standard deviations are presented in S1 Appendix (tables A-D). The quantitative genetic approach cannot test for the presence of both shared environment (C) and dominant genetic (D) effects at the same time due to under-identification. The researcher usually has to choose between ACE models or ADE models. The ratio of MZ/DZ intra-pair correlations can be used as an indicator of the presence of a dominant genetic effect. If the DZ correlation is less than half the MZ, it is an indication of a dominant genetic effect. For a more precise estimation, one can also compare the two models (ACE and ADE) against a saturated model (i.e., model that accounts for all parameters) to assess their respective fit. Another way to consider a small, or negative, DZ correlation relative to the MZ’s is by considering a contrast effect. A contrast effect is assessed with a statistical indicator computed from two regression paths that are fixed to be equal: each stem from one twin to the cotwin. This contrast effect has been interpreted either as indication of a rater bias or an indication of a sibling interaction or competition effect [44]. The rater bias could stem from parent rating twins against each other, systematically rating twins as more dissimilar [5,45,46]. A sibling interaction describes a situation in which each twin influences his cotwin in a dynamic process of continual feedback leading to a greater difference between them. A contrast effect would typically underestimate the DZ resemblance, and thus overestimate the heritability estimate [29].

By looking at the ratio of intra-pair correlations, we suspected dominant genetic effects or contrast effects (see S1 Appendix 1, tables A-D). We tested for the presence of dominant genetic effects (D) in place of shared environment (C) and found that the ACE model with a contrast effect fit better than the ADE. The ACE models used included a contrast effect (parameter S) by the addition of two direct effects between the twins (a first from twin 1 to twin 2 and a second from twin 2 to twin 1). The contrast effects in all ACE models were significant and were necessary components to obtain adequate estimates of heritability of temperamental measures [5,41,47]. In all models, the contrast effects coefficients had a negative value and showed no signs of being affected by zygosity or sex. Thus, the S parameter was set to be equal for MZ and DZ twins, as well as for girls and boys.

In order to further explore sex differences, we used sex-limited models to estimate sex differences in the etiology of negative emotionality [41,42]. We explored the sex differences in cross-sectional models as well as in longitudinal models. The qualitative sex difference analysis aims to determine whether different genes affect each sex (over the ones shared that could also affect with different magnitude), whereas quantitative sex difference analysis determines whether the same genes affect the sexes with different magnitude.

We first tested for qualitative sex differences at each time point to assess whether different sets of genes were associated with the trait for boys and girls. To test for qualitative sex differences, in addition to the ACE factors, a fourth factor (G) is modeled to represent an additive genetic effect specific for boys. The paths to this sex-specific genetic factor were not significant indicating that, for each time point, the same set of genes was influencing both boys and girls. We then executed quantitative sex difference models to assess if the magnitude of the genetic factors were the same for girls and boys. The quantitative sex difference model significantly improved the fit for the 18 months measure, indicating possible sex differences in the magnitudes of genetic effects for girls and boys. As these analyses are important, yet underpowered, these results are presented as exploratory.

Data analyses were performed with Mplus 7.1 [48] using maximum likelihood (ML). All available cases were used for estimation of each parameter. Statistical significance of the individual parameter estimates for the paths in the model was determined by dividing the estimates by their respective standard errors (result is the t-value). The confidence intervals of the A, C, and E variance components were obtained through 10,000 bootstrapped samples.

The goodness of fit was evaluated using Akaike Information Criterion (AIC), the Bayesian Information Criterion (BIC) as well as χ^2^ difference tests comparing twice the difference between the log-likelihood of a model and the saturated model. Degrees of freedom were calculated as the difference in the number of estimated parameters between the two models. A non-significant χ^2^ indicated that the model with fewer parameters fit the data as well as the saturated model and thus had acceptable model fit [49]. For AIC and BIC, a smaller value indicates better fitting models [50,51]. The AIC also reflects the balance between fit and parsimony (fewer parameters; [48]).

#### Longitudinal analyses

Given that the sex-limited model for the 18 month data had the best fit, a sex-limited biometric correlational model was applied to negative emotionality scores at 5 and 18 months [42]. The biometric part of the model allowed for estimates of the additive genetic (A), shared environment (C), and non-shared environment (E) influences respectively at 5 months and 18 months, as well as the correlation between those sources of influences (rA, rC, rE). The correlational approach yields an estimated correlation between two different sets of genes (rA), two different sets of shared environmental effects (rC) and nonshared environmental effects (rE); the higher the correlation, the higher is the “stability” of each effect. Since we were also interested in innovation, we transformed the estimates obtained from the correlational model as if we had executed a Cholesky decomposition using formulas adapted from Loehlin, 1996 [52]. In a Cholesky decomposition, the first factor describes stable (or persistent) effects (genes that are affecting the behaviour at both time points, possibly at different magnitude), while the second factor (and any of the following ones) provides evidence of innovation. Hence, the interpretation in terms of persistence and innovation of genetic and environmental effects and interest in possible sex differences were two reasons to execute a correlational model. As described in Neale (2006) [41], the design of the correlational model that the same set of genes, at a given time point, are used for girls and boys and allow for the magnitude of the effect to vary across sex (i.e. quantitative sex differences; [41]). Statistical significance and goodness of fit tests were performed in the same way as in cross-sectional models.

**Fig 1:**
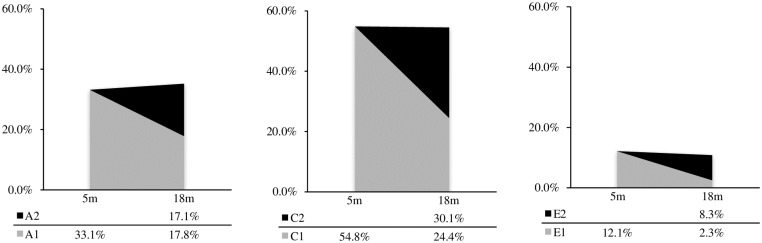
Standardized variance estimates of genetic, shared and nonshared environment latent factors associated with negative emotionality at 5 and 18 months of age. Persistence of effects are indicated by A1, C1 and E1 factors still influencing negative emotionality at 18 months. Innovation effects are indicated by A2, C2 and E2 coming online at 18 months. Percentages add to 100% for each time point.

**Fig 2:**
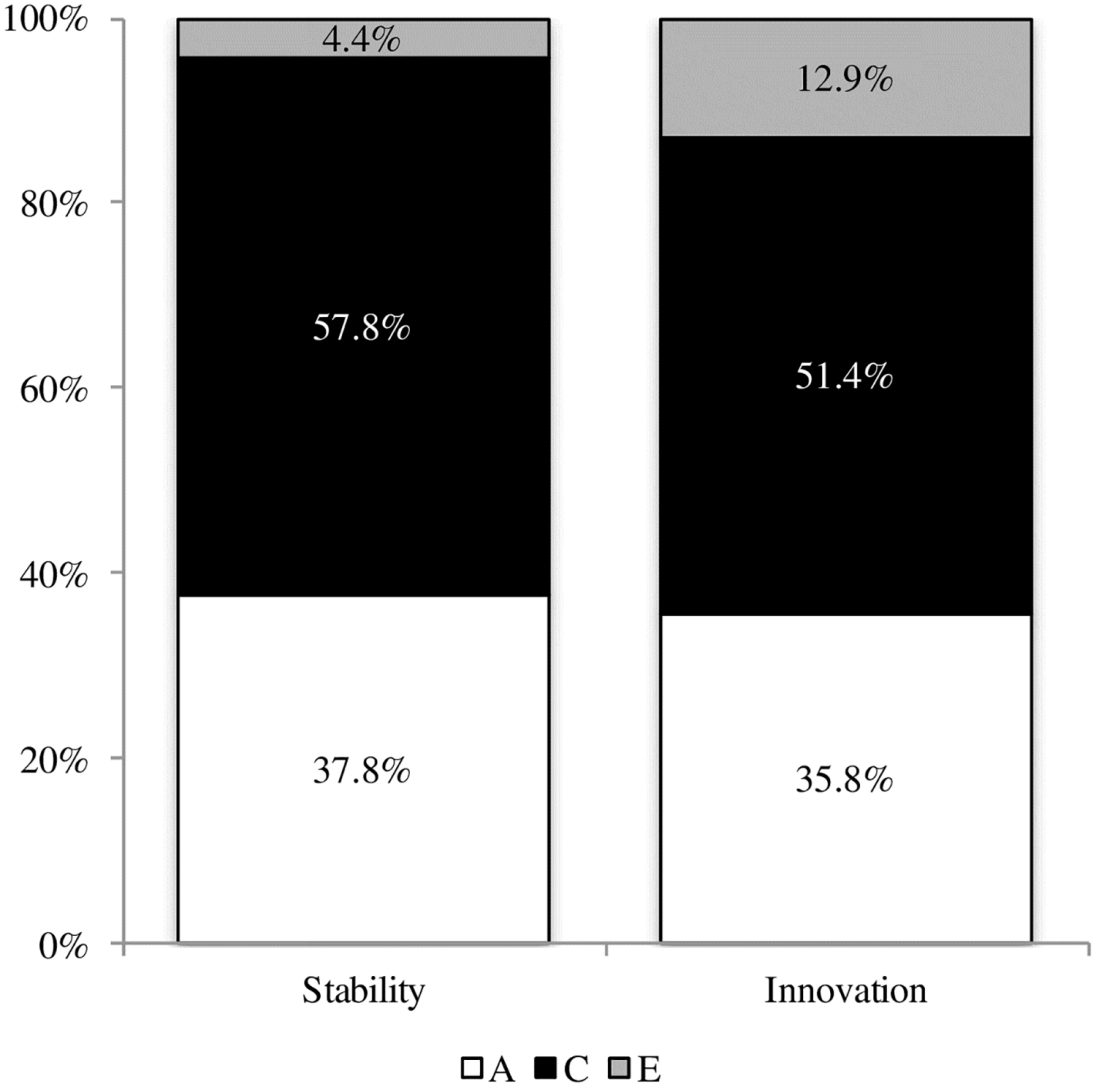
Standardized variance estimates of genetic, shared and nonshared environment latent factors associated with stability of negative emotionality from 5 to 18 months of age and to total innovation effects.

We first computed standardized ACE ratios for each time point (5 months and 18 months) and specified the proportion of each type of effects at 18 months that was explained by influences already present at 5 months (a total of 9 standardized ratios, see Fig 1). The influences on stability of negative emotionality from 5 to 18 months of age as well as innovation effects were also assessed (see Fig 2). The stability of negative emotionality was assessed with the phenotypic correlation that can be obtained from the Cholesky covariance matrix by using standard procedure to compute a correlation from a covariance matrix. For example, with standardized coefficients obtained from a Cholesky decomposition, we can use the following equation *r* = (a_11_*a_21_)+(c_11_*c_21_)+(e_11_*e_21_) to obtain an estimate of the phenotypic correlation [53]. We then computed the proportion of genetic and environmental influences that are part of the phenotypic correlation. To obtain an estimate of genetic influences on the phenotypic stability, we can use (a_11_*a_21_)/*r*.

## Results

### Descriptive statistics

Descriptive statistics for the final sample are in Tables 1 and 2.

**Table 2:** Sample size, means and intraclass correlations of twins at 5 and 18 months separated by sex and zygosity.

Sample size	N of Twin pairs	N of Boys / Girls	MZ pairs m/m	MZ pairs f/f	DZ pairs m/m	DZ pairs f/f	DZ pairs f/m
Negative emotionality at 5 months	598	590 / 606	111	118	94	95	180
Negative emotionality at 18 months	558	543 / 573	105	120	87	87	159
Total sample used for multivariate models	638	634 / 642	119	125	101	99	194
**Means** (SD)			MZ m/m	MZ f/f	DZ m/m	DZ f/f	DZ m/f
Negative emotionality at 5 months			**3.12** (.90)	**3.03** (.98)	**3.16** (1.10)	**3.10** (1.20)	**2.98** (1.10)
Negative emotionality at 18 months			**3.41** (.89)	**3.34** (.86)	**3.32** (1.00)	**3.30** (1.00)	**3.30** (1.10)
Intraclass correlation (based on ANOVA)			MZ m/m	MZ f/f	DZ m/m	DZ f/f	DZ f/m
Negative emotionality at 5 months			.28	.42	-.11	-.05	-.10
Negative emotionality at 18 months			.54	.31	-.18	.14	-.12

Note: The full correlation matrix is presented in Appendix. MZ = monozygotic twins, DZ = dizygotic twins, f/f = female/female twins, m/m = male/male twins, f/m = female/male twins, SD = standard deviation

### Univariate results

#### 5 months

The magnitude of genetic and environmental effects was similar for boys and girls at 5 months. Models constraining the correlations among ACE latent variables to be equal across sex fit equally well as unconstrained models, suggesting no significant sex difference. The ACE models without sex differences and with contrast effects exhibited indices of acceptable fit (Table 3).

**Table 3:** Comparison of fit for univariate and multivariate models (with contrast effect).

Model	Log likelihood	χ^2^ (df)	*p*	AIC	BIC
*5 months* (*N* = 1196)					
1. Saturated	-1711.73			3457.46	3532.15
2. ACE sex-limited	-1715.37	7.27 (8)	.51	3448.74	3488.28
3. ACE no sex limitation	-1716.08	8.70 (11)	.65	3444.16	3470.52
*18 months* (*N* = 1116)					
1. Saturated	-1520.94			3075.87	3149.38
2. ACE sex-limited	-1524.41	6.94 (8)	.54	3066.81	3105.73
3. ACE no sex limitation	-1527.459	13.05 (11)	.29	3066.92	3092.86
*Multivariate*					
1. Saturated	-3152.84			6397.69	6602.77
2. ACE sex-limited	-3163.50	21.33 (26)	.72	6367.01	6456.18
3. ACE no sex-limitation	-3168.48	31.27 (32)	.50	6364.95	6427.37

AIC = Akaike information criterion, BIC = Bayesian information criterion

Standardized parameter estimates, confidence intervals, and variance proportions for the 5 month model are presented in Table 4. At 5 months, the variability estimates in negative emotionality attributed to genetic, shared environmental, and unique environmental influences were 24%, 67%, and 9%, respectively, for both sexes. Both sexes demonstrated substantial shared environmental influence and genetic influence.

**Table 4:** Longitudinal standardized parameter estimates, confidence intervals, and variance proportions.

	Parameter estimates and 95% confidence interval	Correlations	Contrast effect	Variance proportions (standardized)
5 month model (with contrast effect, no sex limitation)	a_1_ = .60 (0.03–0.95)		s_1_ = −.52	a = .24
	c_1_ = .99 (0.00–1.51)			c = .67
	e_1_ = .36 (0.01–0.61)			e = .09
18 month model (with contrast effect, no sex limitation)	a_1_ = .57 (0.04–0.94)		s_1_ = −.52	a = .25
	c_1_ = .94 (0.00–1.40)			c = .67
	e_1_ = .32 (0.01–0.53)			e = .08
Longitudinal model (with contrast effect)	a_11_ = .68 (0.21–0.89)	*r*_e_ = .15	s_1_ = s_2_ = −.46	(See Fig 1)
	a_21_ = .28 (0.05–0.48)	*r*_a_ = .42		
	a_22_ = .60 (0.19–0.78)	*r*_c_ = .49		
	c_11_ = .88 (0.45–1.4)			
	c_21_ = .40 (0.13–0.62)			
	c_22_ = .71 (0.24–1.2)			
	e_11_ = .41 (0.13–0.57)			
	e_21_ = .06 (-0.00–0.13)			
	e_22_ = .36 (0.11–0.49)			

Caption: The parameter estimates were standardized for the computation of ACE proportions of variance estimates.

#### 18 months

The likelihood ratio test showed that the fit was slightly improved in models with sex constraints, but other fit indices (AIC and BIC) indicated that models without sex limitation had better fit. Hence it was more parsimonious to accept the model without sex differences as the final 18-month model. A contrast effect also improved the fit of the model (Table 3). At 18 months, twins demonstrated similarly substantial shared environmental influence and genetic influence as they had at 5 months (Table 4). The univariate results are so similar to the multivariate ones that we only discuss the latter in the following section.

### Longitudinal analyses

**Fig 3:**
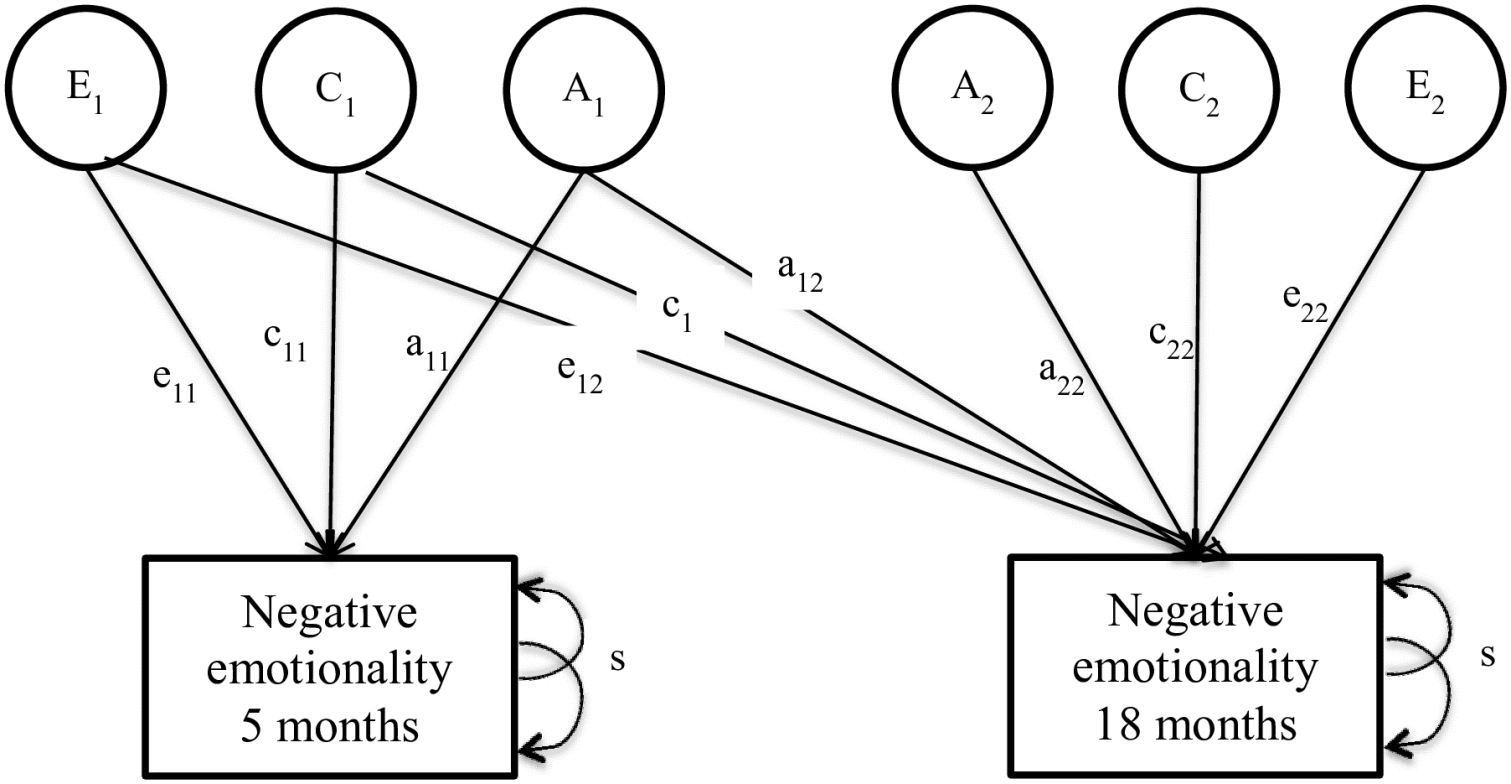
Theoretical path diagram: Biometric Cholesky decomposition. Persistence effects are denoted by the subscript 12; innovation effects are denoted by the subscript 22.

Longitudinal data were analyzed with a biometric correlational model (N = 1276 or 638 pairs) to evaluate the persistence and innovation of gene-environment’s influence on negative emotionality scores at 5 and 18 months of age as well as on interindividual stability between the two time points. A saturated model was used as a baseline to which the constrained models were compared. Contrast effect parameters were estimated and set to be equal between girls and boys as well as between MZ and DZ pairs because the fit was not improved by specifying different values. Likelihood ratio test and BIC indicated a somewhat adequate fit for the model with sex constraints, but AIC indicated that the model without sex limitation had a better fit (see Table 3). The sex-limited model showed interesting preliminary results: girls’ innovation effects were attributed to genetic (29.9%) and shared environment (42.2%) factors, whereas innovation effects for boys were genetically mediated (76.3%). However, we lacked the power required to detect sex effects. In the interest of parsimony, the model without sex limitation was accepted as the final model. Genetic and environmental contributions to each time point as well as their persistence are shown in Fig 1, while influences on stability and total innovation effects have been computed as described in above methods and are illustrated in Fig 2. The path diagram corresponding to a Cholesky decomposition is illustrated in Fig 3 and the corresponding parameter estimates (transformed from the correlational model) for the genetic, shared environment and nonshared environment are detailed in Table 4. The correlations between the genetic, shared environment and nonshared environment latent factors as well as the contrast effect are also shown in Table 4.

At both 5 and 18 months, estimates of the genetic and environmental contributions to negative emotionality were almost identical. Heritability accounted for approximately 34% of the variance in negative emotionality, showing substantial genetic transmission. Shared environmental influences explained 54% of the variance in this trait.

The extent to which the trait presented interindividual stability over time was due to both genetic (37.8%) and shared environment (57.8%) factors. Innovation effects were similarly mediated, with the proportions of innovation effects attributed to genetic and shared environmental factors at 35.8% and 51.4%, respectively.

## Discussion

The current research used a large sample of twin participants followed from birth in order to examine the persistence and innovation in the genetic and environmental sources of variance of temperamental negative emotionality in the first 2 years of life. Importantly, the longitudinal approach allowed for an evaluation of the gene-environment underpinnings of the phenotypic stability in this trait. We found that the ACE model with contrast effect (possibly a rater bias) fit our data best. Within each time point, we found substantial heritable (approximately 34%) and shared environmental (54%) contributions to negative emotionality. As well, both genetic and shared environmental influences mediated the stability in the trait and presented a dynamic influence pattern. Notably, we saw evidence of both genetic and environmental innovative effects coming online at 18 months. These results suggest that in infancy, genetic and environmental influences support the stability of temperamental negative emotionality and that those influences are also dynamic.

The magnitude of heritability is substantial and commensurate with the low end of previous research on temperament (between 40% and 48%). While some similar studies have found a negligible contribution of the shared environment and found an AE model as best fit [2,5,27], the present study evidenced a substantial shared environment influence. The final multivariate ACE model with a contrast effect, but invariant with respect to sex, was consistent with the two similar longitudinal twin studies [10,29].

However, contrary to previous research [9,21,30,54], the contributions of genetic and environmental factors did not change between 5 and 18 months. The contrast in these findings could be the result of different methodology engaging different processes and measuring different constructs of temperament or behaviour [54]. The longitudinal consistency of proportions attributed to genetic and environmental factors result indicates some persistence in these influences in early infancy.

Furthermore, our results indicated that genetic and shared environmental factors account for why young children stay on course with respect to negative emotionality in the first two years. The finding that genetic effects show persistence over time is consistent with the longitudinal twin study literature on negative emotionality [9]. The persistence of the shared environment effect over time is a more novel result and suggests that the environment plays a larger role in the development of temperament in early infancy than previously estimated.

Similarly, the innovation effect on negative emotionality was mediated by both genetic and shared environmental influences. These substantial genetic innovations are somewhat dissimilar to Rhee et al.’s [9] observation that age-specific influences were mostly limited to non-shared environmental influences. However, there is evidence that new genetic effects begin to shape childhood outcomes of negative emotionality such as affect and adaptability [29,54], as well as physical [34] and reactive aggression [55]. For example, the innovation effect we observed in negative emotionality at 18 months could represent genetically-driven maturation or age-related increases in socio-cognitive development, such as the emergence of social communication [54]. As well, the timing and influence of new genetic factors coming online during development could be programmed, in part, by environmentally-driven epigenetic mechanisms [56]. Thus, we observed that negative emotionality was developmentally dynamic, and that these new genetic contributions may also be accompanied by increasing susceptibility to shared environmental factors [35]. In this way, heritable and environmental factors are responsible for stability, and present a dynamic influence pattern.

Despite limited power, our preliminary results suggest possible sex differences (i.e., temperament was increasingly associated with genetic factors for boys, while the shared environment continued to have a significant effect on girls’ temperament) that need to be confirmed with larger samples.

This research has limitations that may affect its generalizability. First, we employed single informant measures of temperament of twin infants. A mother could systematically rate her twins differently comparing one to another, which could have led to a rater bias and contributed to the results (tends to inflate heritability estimates; [5,28]). We minimized such effects by having each member of the pair rated at a different moment in time (approximately two weeks apart). Second, our results were based on a sample recruited in a specific cultural context, and thus may not generalize to all cultural contexts. Regardless, the present research was carried out with a large, relatively diverse sample, which was very similar in terms of almost all demographic characteristics as compared to the full cohort.

Further examination of how the environment moderates development of temperament in young children and the developmental timing of key periods for this process to occur are important next steps for research in this area. For example, Lemery-Chalfant and colleagues [3] found that heritability of negative affectivity increased under crowded or unsafe home conditions, exemplifying the complex and influential interaction between genetic and environmental factors on temperament.

To conclude, the current research showed that in the first two years of life, both genetic and shared environmental factors contribute substantially to negative emotionality. The trait’s persistence across time appears to be both genetically- and environmentally- mediated. Furthermore, evidence of innovative effects indicated that negative emotionality is developmentally dynamic and affected by new genetic and environmental factors at 18 months. The present study’s large sample size, longitudinal design, and measures in infancy offer more precise estimates of genetic and environmental contributions, to trait negative emotionality. The results of the present study stand to further improve prevention and intervention programs aimed to reduce mental health problems in high-risk populations. For instance, it has widely been demonstrated that difficult temperament is a predictor of mental health problems such as depression, anxiety, and aggression in childhood [57,58] or adulthood [7,8,12]. The substantial environmental influences in the first two years of life support the need for early interventions at a very early stage; i.e. during pregnancy or in the first years of life [59].

## Supporting information

S1 Appendix Correlation matrices with means and standard deviations. (DOCX)
