# Supplementary material for: Persistence and innovation effects in genetic and environmental factors in negative emotionality during infancy: A twin study
Source: PLoS One. 2017 Apr 27;12(4):e0176601. doi: 10.1371/journal.pone.0176601 (PMC5407782; doi:10.1371/journal.pone.0176601)
Supplement: S1 Appendix — (DOCX) [file pone.0176601.s001.docx]

**Appendix S1 Correlation matrices with means and standard deviations**

Table A

*All sample: MZ / DZ correlation matrices with means and standard deviations*

|  |  | DZ pairs  N=394 |  | |  |  |  |  |  |  |  |
| --- | --- | --- | --- | --- | --- | --- | --- | --- | --- | --- | --- |
|  | MZ pairs  N=244 |  | Twin 1 | |  | Twin 2 | |  | Mean (DZ) | | SD (DZ) |
|  |  |  | 5 m | 18 m |  | 5 m | 18 m |  |  | |  |
| Twin 1 | | 5 m | - | 0.381 |  | -0.078 | 0.023 |  | 2.954 | | 1.069 |
|  | 18 m | | 0.333 | - |  | 0.085 | -0.062 |  | 3.213 | | 1.006 |
|  |  |  |  |  |  |  |  |  |  | |  |
| Twin 2 | | 5 m | 0.369 | 0.272 |  | - | 0.385 |  | 3.176 | | 1.150 |
|  | 18 m | | 0.216 | 0.421 |  | 0.333 | - |  | 3.395 | | 1.075 |
|  |  |  |  |  |  |  |  |  |  | |  |
| Mean (MZ) | |  | 3.019 | 3.350 |  | 3.130 | 3.393 |  |  | |  |
| SD (MZ) | |  | 0.915 | 0.826 |  | 0.978 | 0.911 |  |  | |  |

Note: MZ = monozygotic twins, DZ = dizygotic twins, SD = standard deviation

Table B

*Same-sex pairs of boys MZ / DZ correlation matrices with means and standard deviations*

|  |  | DZ pairs  N=101 |  | |  |  |  |  |  |  |  |
| --- | --- | --- | --- | --- | --- | --- | --- | --- | --- | --- | --- |
|  | MZ pairs  N=119 |  | Twin 1 | |  | Twin 2 | |  | Mean (DZ) | | SD (DZ) |
|  |  |  | 5 m | 18 m |  | 5 m | 18 m |  |  | |  |
| Twin 1 | | 5 m | - | 0.316 |  | -0.100 | -0.030 |  | 3.062 | | 1.084 |
|  | 18 m | | 0.366 | - |  | -0.060 | -0.174 |  | 3.278 | | 0.990 |
|  |  |  |  |  |  |  |  |  |  | |  |
| Twin 2 | | 5 m | 0.306 | 0.350 |  | - | 0.378 |  | 3.316 | | 1.139 |
|  | 18 m | | 0.212 | 0.543 |  | 0.338 | - |  | 3.350 | | 0.994 |
|  |  |  |  |  |  |  |  |  |  | |  |
| Mean (MZ) | |  | 3.013 | 3.317 |  | 3.202 | 3.444 |  |  | |  |
| SD (MZ) | |  | 0.922 | 0.857 |  | 0.883 | 0.915 |  |  | |  |

Table C

*Same-sex pairs of girls MZ / DZ correlation matrices with means and standard deviations*

|  |  | DZ pairs  N=99 |  | |  |  |  |  |  |  |  |
| --- | --- | --- | --- | --- | --- | --- | --- | --- | --- | --- | --- |
|  | MZ pairs  N=125 |  | Twin 1 | |  | Twin 2 | |  | Mean (DZ) | | SD (DZ) |
|  |  |  | 5 m | 18 m |  | 5 m | 18 m |  |  | |  |
| Twin 1 | | 5 m | - | 0.373 |  | -0.035 | -0.056 |  | 2.930 | | 1.022 |
|  | 18 m | | 0.300 | - |  | 0.222 | 0.170 |  | 3.163 | | 0.901 |
|  |  |  |  |  |  |  |  |  |  | |  |
| Twin 2 | | 5 m | 0.429 | 0.214 |  | - | 0.349 |  | 3.270 | | 1.285 |
|  | 18 m | | 0.222 | 0.310 |  | 0.323 | - |  | 3.454 | | 1.137 |
|  |  |  |  |  |  |  |  |  |  | |  |
| Mean (MZ) | |  | 3.027 | 3.378 |  | 3.065 | 3.348 |  |  | |  |
| SD (MZ) | |  | 0.907 | 0.796 |  | 1.056 | 0.906 |  |  | |  |

Table D

*Opposite sex DZ pairs correlation matrices with means and standard deviations (N=194 pairs)*

|  |  |  |  | |  |  |  |
| --- | --- | --- | --- | --- | --- | --- | --- |
|  |  |  | Girls | |  | Boys | |
|  |  |  | 5 m | 18 m |  | 5 m | 18 m |
| Girls | | 5 m | - |  |  |  |  |
|  | 18 m | | 0.411 | - |  |  |  |
|  |  |  |  |  |  |  |  |
| Boys | | 5 m | -0.107 | 0.081 |  | - |  |
|  | 18 m | | 0.102 | -0.120 |  | 0.424 | - |
|  |  |  |  |  |  |  |  |
| Mean (DZO) | |  | 2.906 | 3.207 |  | 3.057 | 3.389 |
| SD (DZO) | |  | 1.078 | 1.066 |  | 1.065 | 1.083 |
